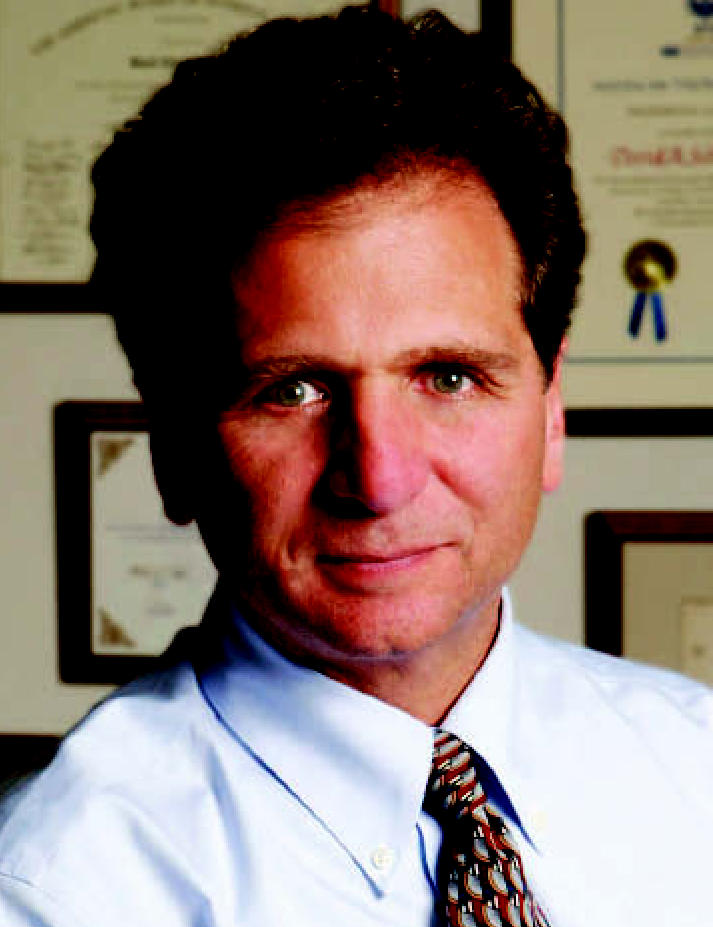# A New Venue for the Director’s Perspective

**DOI:** 10.1289/ehp.115-a182

**Published:** 2007-04

**Authors:** David A. Schwartz

**Affiliations:** Director, NIEHS and NTP, E-mail: david.schwartz@niehs.nih.gov

Two years ago this month I began writing this column for *EHP*. As the new NIEHS director, my goal for this column was to maximize communication with the environmental health sciences community about my vision, goals, and strategy for the institute, as well as convey information and opinion on topics I felt were important to the field. Although it seemed to me at the time that my use of *EHP* as a forum was a natural and reasonable activity given the journal’s leadership and readership in environmental health sciences, time and experience have led me to a new recognition that this might be viewed as undue editorial influence over the journal’s content. I am fully committed to ensuring the scientific credibility of *EHP*, for which editorial independence is vital, and thus have decided to remove the Director’s Perspective column from the journal and relocate it to a more appropriate venue on the NIEHS website.

*EHP* ’s relationship with the NIEHS has always been somewhat unique. Although many government-supported publications and even scientific journals exist, few have been supported directly by the NIH. *EHP* was begun as an NIEHS-supported journal in 1972 to fill a much-needed gap in scientific publishing in the fledgling field of environmental health sciences. And in fact, it has been the continued strong support of the NIEHS and the outstanding work of a relatively small group of highly dedicated individuals that have allowed the journal to evolve into the preeminent environmental sciences journal in the world. The journal was established with the tacit understanding that the editorial guidance of *EHP* would have to function independently in substance for the journal to succeed and serve its purpose, and safeguards including an outside, independent editorial board and appropriate scientific peer-review policies—and with later expansion, expert and outside review of news content and a stringent competing financial interest policy—were put into place, and continue to guide the journal’s practices today.

But the scientific climate and public concerns about real and perceived conflicts of interest have changed over the past 35 years. Whereas in the past these safeguards, along with the personal integrity of the editorial leadership, may have been enough to convey the integrity of the journal, in today’s world more is expected. Today, it is not necessarily sufficient for a publication to be true in substance; it must also be true in appearance.

In point of fact, *EHP* has functioned for decades with the utmost editorial freedom while enjoying the strong financial support of the NIEHS. With *EHP* being as the world’s leading journal in the field of environmental health sciences, it is entirely reasonable that the content of *EHP* and the activities and goals of the NIEHS often overlap; in fact it would be ludicrous if they did not. However, support and influence are not easily separated in appearance. Because scientific journals rise and fall on the basis of their perceived credibility, I believe the NIEHS has a clear responsibility to ensure that *EHP* continues to be viewed as entirely credible in publishing the very best research in our field. While the NIEHS has made a commitment to continue to strongly support *EHP* financially, we are taking new measures to ensure the journal’s editorial independence and to increase the transparency of this feature to the scientific community and the public.

One way in which we are accomplishing this goal is through the recruitment of a new editor-in-chief for *EHP* who will reside outside the NIEHS. The person we are looking for will be an accomplished scientist and a thought leader in the field who, supported by a strong and active editorial board, will provide independent scientific and editorial leadership for the journal. The capacity of the editorial board to provide input into *EHP* policies and practices, as well as its everyday impact on the scientific content of the journal, will be enhanced through more regular board meetings and improved methods to handle and review manuscripts. Although the NIEHS News section will be moved from *EHP* to the new NIEHS website upon its launch in June 2007, our outstanding science news coverage will be maintained in *EHP*, albeit reduced in scope. And although I may occasionally in the future submit scientific or editorial content to *EHP*, such pieces will undergo standard peer review and editorial consideration prior to acceptance for publication. I truly believe that everything published in *EHP* should be the decision of the editorial staff, and not influenced by the source of the journal’s funding.

I also continue to believe emphatically in the importance of my personally communicating with the environmental health sciences community, and will take advantage of the new venues and technologies that soon will become available through the NIEHS’s transformed website to do so, starting with creating a home for the Director’s Perspective. These columns allow me to let you know what I’m thinking and how I plan to address the ongoing challenges facing our institute and our field. However, it’s important for me to hear and understand your thoughts and concerns as well. I’m hoping that our new website will create opportunities for us to interact in innovative ways that allow us to share ideas and build consensus. Approaches such as e-mail, instant messaging, surveys, blogs, and Wikipedia-like discussion sites may facilitate the open and interactive process that I believe is critically important to the future of environmental health sciences.

Independence and transparency are cornerstones of science—and of scientific publishing. It is my hope that with enhancements to both of these, the NIEHS and *EHP* will continue to play vital roles in furthering environmental health sciences to the betterment of human health and well-being.

## Figures and Tables

**Figure:**